# Implications of RNA Viruses in the Male Reproductive Tract: An Outlook on SARS-CoV-2

**DOI:** 10.3389/fmicb.2021.783963

**Published:** 2021-12-24

**Authors:** Mohammad Ishraq Zafar, Jiangyu Yu, Honggang Li

**Affiliations:** ^1^Institute of Reproductive Health/Center of Reproductive Medicine, Tongji Medical College, Huazhong University of Science and Technology, Wuhan, China; ^2^Department of Reproductive Medicine, Central Theater Command General Hospital of Chinese People’s Liberation Army, Wuhan, China; ^3^Wuhan Tongji Reproductive Medicine Hospital, Wuhan, China

**Keywords:** RNA virus, SARS-CoV-2, COVID-19, male infertility, sexual transmission, seminal fluid

## Abstract

Emerging viral infections continuously pose a threat to human wellbeing. Several RNA viruses have managed to establish access to the male reproductive tract and persist in human semen. The sexual transmission of the virus is of critical public concern. The epidemiological inferences are essential to understand its complexity, particularly the probability of viral transmission from asymptomatic patients or those in the incubation period or from the patient who was previously infected and now fully recovered. From the clinical perspective, negative impacts in the male reproductive tract associated with RNA virus infection have been described, including orchitis, epididymitis, impaired spermatogenesis, and a decrease in sperm quality, which can affect male fertility at different time intervals. The disruption of anatomical barriers due to inflammatory responses might enable the viral invasion into the testis, and the immune privilege status of testes might facilitate a sustained persistence of the virus in the semen. In this review, the current knowledge about other RNA viruses that affect male reproductive health provides the framework to discuss the impact of the SARS-CoV-2 pandemic. The molecular mechanisms, sexual transmission, and viral impacts for mumps, HIV, Zika, and Ebola viruses are explored. We discuss the currently available information on the impact of SARS-CoV-2 and its sequelae in the male reproductive tract, particularly regarding presence in semen, its impact on sexual organs, and sperm quality. To date, no sexual transmission of SARS-CoV-2 has been reported, whereas the identification of viral particles in semen remains conflicting. In the purview of the earlier conducted analyses, it is essential to investigate further the long-term health impacts of SARS-CoV-2 on the male reproductive tract.

## Introduction

The global pandemic caused by the outbreak of the severe acute respiratory syndrome coronavirus 2 (SARS-CoV-2) (Wang C. et al., 2020; Zhou P. et al., 2020) has caused 250 million infections and 5 million deaths worldwide^1^. Intense efforts have been made to identify viral transmission pathways to contain the pandemic and to understand the clinical consequences of the patients. The transmission of SARS-CoV-2 occurs primarily through respiratory droplets and aerosols generated during coughing or sneezing (Zhou et al., 2021).

In addition, the viral RNA has been identified in biological samples, including blood, urine, and feces (Paoli et al., 2020; Xie et al., 2020), so it is critical to explore whether alternative modes of transmission of the virus are possible.

SARS-CoV-2 uses angiotensin-converting enzyme 2 (ACE2) as a “gate” for invading cells. In addition, Transmembrane serine protease 2 (TMPRSS2) enhances ACE2-mediated virus entry (Walls et al., 2020). Both ACE2 and TMPRSS2 proteins are expressed in the testis and male reproductive tract (Hamming et al., 2004; Jia et al., 2005; Hikmet et al., 2020; Lee et al., 2020; Song H. et al., 2020; Wang and Xu, 2020). If the virus can infect human testes, it may affect multiple pathways, reduce male fertility, and even potentially involve sexual transmission and, consequently, risk of vertical transmission, embryonic infections, and congenital diseases (Salam and Horby, 2017).

The isolation of viruses from male reproductive organs is not new, especially from the semen of infected male patients. Testes are immunologically privileged sites, but certain viruses can breach and cross the blood–testis barrier. In human semen, nearly 27 viruses have been identified to date (Salam and Horby, 2017), including several RNA viruses. Most of the infections can cause direct deleterious effects on the spermatozoa, sperm production, testis function, and other reproductive tract organs that could cause male infertility in 50% of the cases (Dejucq and Jégou, 2001; Pike et al., 2021).

The virus may persist following an acute infection, and theoretically, it can replicate within the male reproductive tract.

This review discusses the survival and distribution of RNA viruses in the male reproductive system and its implications clinically. The role of the immune-privileged nature of testis and the function of the blood–testis barrier in disseminating infectious viral particles are explored. The current review aims to consolidate and discuss the current information concerning the SARS CoV-2 virus’ impact on the male reproductive tract, shed light on viral presence in semen, and emphasize the challenges posed to male reproductive health during and after the pandemic.

## Physiological Organization and Barriers for RNA Viruses in the Male Reproductive System

**FIGURE 1:**
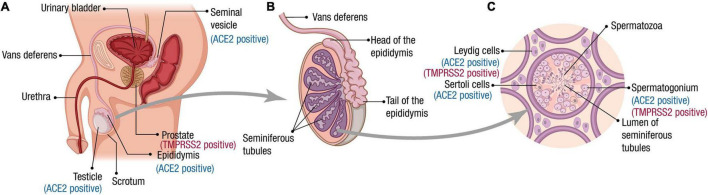
Male reproductive tract, spermatogenesis, and SARS-CoV-2 receptor expression. **(A)** Scheme of the male reproductive system composition. The organs for which ACE2 and TMPRSS2 expression has been reported at RNA and/or protein level are indicated. **(B)** The testes are composed mainly by seminiferous tubules (up to 90% of the testis volume). Immotile sperm flow from the lumen of the seminiferous tubules into the epididymis via the rete testis. During the passage through the epididymis to the vas deferens, the spermatozoa mature and acquire their motility. **(C)** A transverse section of seminiferous tubules is illustrated. The cells for which ACE2 and TMPRSS2 expression has been reported at the RNA and/or protein level are indicated. Spermatogenesis occurs within the seminiferous tubules. Spermatogonia (diploid primordial germ cells) are located near the basal lamina of the seminiferous tubules. When spermatogonia divide by a first mitotic division, they produce diploid primary spermatocytes. Half of these primary spermatocytes will remain near the basal lamina dividing mitotically to allow the production of new spermatozoa during the whole male’s reproductive lifespan. The other half migrate toward the lumen of the seminiferous tubules and undergo meiosis I, generating haploid secondary spermatocytes, which divide through meiosis II to produce haploid spermatids. A final stage called spermiogenesis produces mature sperm cells (spermatozoa) capable of fertilizing an egg. Leydig cells produce the hormone testosterone.

The male reproductive system is essential for the maintenance of the species for all sexual organisms. In mammals, testes are responsible for producing spermatozoa and the synthesis of hormones, which take place in two compartments morphologically and functionally distinguishable from each other but are closely connected (Ilacqua et al., 2017). Spermatogenesis is performed in the seminiferous tubules that contain the germ cells and two different types of somatic cells, the peritubular cells, and the Sertoli cells (Figure 1). The interstitial compartment between the seminiferous tubules contains the Leydig cells, source of testosterone, and insulin-like factor 3 (INSL3).

An epididymis covers the head of each testis, a tightly coiled tube connecting the efferent ducts to the vas deferens, where the sperm mature and are stored (Figure 1). Once out of the epididymis, the sperm pass through the ductus deferens mixing with fluids from associated seminal vesicles, which contain vast amounts of sugar for ATP generation and movement of sperm in the female reproductive tract (Ilacqua et al., 2017). Once the sperm and seminal vesicle secretions are mixed, this seminal fluid is transported to the prostate gland, which secretes an alkaline and milky fluid to the seminal mixture passing through it, causing the thickening of the semen after ejaculation. Finally, the semen is ejaculated from the penis through the urethra (Revenig et al., 2014).

### The Blood–Testis Barrier

In the mammalian body, the most secured blood–tissue barrier is the blood–testis barrier (BTB) (Mruk and Cheng, 2015), dividing seminiferous epithelium into two compartments, namely, the basal and apical (adluminal) compartments. During their development, germ cells are displaced from the basal to the adluminal compartment. The BTB renders an impermeable physical barrier that sets apart the events of spermatogenesis; the basal compartment of the epithelium is just outside of the BTB, where the differentiation and self-renewal of spermatogonia and the progression of the cell cycle until preleptotene spermatocyte take place. In the apical compartment located behind the BTB, meiosis (I and II), spermiogenesis, and spermiation take place (Ilacqua et al., 2017).

In mammalian testes, the BTB has specialized junctions–tight junctions, basal ectoplasmic specialization, gap junction, and desmosomes–between adjacent Sertoli cells in the seminiferous epithelium. The BTB is a dynamic structure. This is accomplished by synchronizing dissolution and reassembly of the tight junctions above and below the migrating germ cells. The blood vessels, lymph vessels, or nerves are identified between the interstitium tubules and do not penetrate the seminiferous tubule (Cheng and Mruk, 2012).

Two essential functions are postulated for the BTB (Mruk and Cheng, 2015). First, the immune system recognizes the physical isolation of haploid cells as antigenic and denies access of antibodies and immune cells into the tubule. During meiosis and spermiogenesis, some germ cell–specific antigens (proto-oncogenes and oncogenes) are expressed, which could cause infertility by eliciting an immunological reaction and autoimmunity. Second is the preparation of a particular milieu for the meiotic process and sperm development. Along the cell body, extending over the entire height of the germinal epithelium, all morphological and physiological differentiation and maturation of the germinal cell up to the mature sperm cell occur. The limited access to the secretory activity of Sertoli cells in and out of the lumen and the difference in composition of tubular and interstitial fluid creates a uniquely nurturing environment for the developing germ cells (Fijak and Meinhardt, 2006).

In addition to BTB, the epididymal lumen is also an immune-privileged or immune-protected site for sperm (Gregory and Cyr, 2014). Tight junctions between adjacent epithelial cells of the epididymis create a blood–epididymis barrier (BEB) that keeps checking on the exchange of the molecules between blood and lumen. This barrier, along with selective transport by principal cells, enables the epididymis to concentrate essential organic molecules required for the sperm maturation and avoids an autoimmune reaction (immunogenic) against sperm (Dufour et al., 2007).

Therefore, for both the BTB and BEB, the interplay of the three components (anatomic, physiologic, and immunologic) makes the barriers potent *per se*. Several integral membrane proteins contribute to the barrier, and fence function of tight junctions; each of these proteins plays an essential role in the movement of viral and cancer cells across the barrier (Mruk and Cheng, 2010).

## RNA Viruses and the Male Reproductive Tract

Over the years, at least 27 viruses, including several RNA viruses, have been identified in humans’ semen and reproductive tract. These viruses come from diverse families (Salam and Horby, 2017), suggesting that viruses in the male genital tract are not exclusively dependent on a specific or conserved viral epitope or a shared mechanism of immune evasion. Many of the viruses identified in the male reproductive system are RNA viruses (Liu et al., 2018), so particular emphasis on the impact of these viruses on male fertility and sexual transmission is done in the context of the SARS-CoV-2 pandemic (Table 1).

**TABLE 1:** Major RNA viruses found in the male reproductive tract, their consequences, and cell receptors used for viral entry.

RNA virus	Family	Main impact in the male reproductive tract reported	Virus shedding in human semen	Main cell receptor	References
HIV	*Retroviridae*	Orchitis, infertility, sexual transmission	Acute stage: 61–100% Chronic stage: 81–100%	CD4	Dejucq and Jégou, 2001; Salam and Horby, 2017; Liu et al., 2018; Le Tortorec et al., 2020
Zika virus (ZIKV)	*Flaviviridae*	Orchitis, infertility, risk of sexual transmission	Acute stage: 50–68%	AXL receptor	Musso et al., 2015; Mead et al., 2018; Strange et al., 2019; Le Tortorec et al., 2020
Hepatitis viruses C	*Flaviviridae*	Alteration of sperm parameters.	Acute stage: 29–39% Chronic stage: 32–46%	NA	Liu et al., 2018; Le Tortorec et al., 2020
Mumps virus (MuV)	*Paramyxoviridae*	Orchitis, testicular atrophy, infertility	NA	Sialic acid, AXL, and MER receptor tyrosine kinases	Dejucq and Jégou, 2001; Liu et al., 2018
Ebola virus (EBOV)	*Filoviridae*	High risk of sexual transmission.	Acute stage: 73–100%	Different molecules reported, including integrins, C-type lectins, and AXL	Soka et al., 2016; Deen et al., 2017; Perry et al., 2018; Schindell et al., 2018; Le Tortorec et al., 2020
Influenza virus	*Orthomyxoviridae*	Orchitis.	NA	Sialic acid	Dejucq and Jégou, 2001; Liu et al., 2018
SARS-CoV	*Coronaviridae*	Orchitis.	NA	ACE2	Zhao et al., 2003; Xu et al., 2006
SARS-CoV-2	*Coronaviridae*	Testicular damage, orchitis, epididymitis, impaired spermatogenesis	Only two reports among many studies detected viral RNA in semen and in 6–15%% of the patients	ACE2	Li D. et al., 2020; Li H. et al., 2020; Paoli et al., 2020; Ma et al., 2021

The male reproductive system infection may take place in the context of viremia because the BTB and BEB are imperfect barriers to viruses, especially during systemic or local inflammation (Li et al., 2012). Moreover, because the testes are immunologically privileged to preserve fertility, viruses could persist longer in the semen than other body fluids, even incapable of replication out from the surveillance of the immune system (Epelboin et al., 2017).

Any human male reproductive tract organ can be infected, but the testicular and epididymal inflammatory conditions are more commonly observed than other accessory glands (Liu et al., 2018). The persistence and survival of RNA viral particles in semen have been accounted as the main reason for sexual transmission of viruses such as the flaviviruses Zika (ZIKV) and hepatitis B (HBV), filoviruses like Ebola (EBOV), and retroviruses like HIV (Table 1).

### Orchitis and Testis Affections Caused by RNA Viruses

The human immunodeficiency virus (HIV) and mumps virus (MuV) are the most popular and broadly studied viruses that cause testicular disorders (Table 1). Orchitis is an inflammation of the testis involving single or both testicles due to the hematogenous propagation of the pathogens (Schuppe et al., 2008; Liu et al., 2018). It is characterized by leukocyte infiltration into the seminiferous tubules leading to cellular and tubular damage. Orchitis may affect Sertoli and Leydig cells and disrupt the immune-privileged environment. It may affect male infertility because of the loss of a protective environment, regarded as necessary for sperm maturation and the production of reproductive hormones, which may affect male fertility (Ludwig, 2008; Schuppe et al., 2008; Olaniyan et al., 2020).

Mumps is the most common cause for viral orchitis (Dejucq and Jégou, 2001; Masarani et al., 2006). Orchitis affects 20–30% of adult mumps patients, being the most common clinical complication. Mumps orchitis frequently occurs 4 to 10 days after the salivary glands swell (parotitis). Of affected testicles, 30–50% demonstrate a degree of testicular atrophy. Unilateral involvement is more common in patients with orchitis, whereas bilateral involvement accounts for 15 to 30% (Wu et al., 2021). In the earlier days of infection, the virus attacks the testicular glands, leading to parenchymal inflammation, lymphocyte infiltration, and seminiferous tubule damage (Wu et al., 2019). The MuV vaccine administration in children is highly effective in reducing the incidence of mumps. However, the increased frequency of orchitis due to recent global outbreaks has been considered a threat to male fertility (Masarani et al., 2006). Mumps orchitis can affect Leydig cells causing low testosterone levels, elevated luteinizing hormone levels, and imbalanced pituitary feedback regulation to luteinizing hormone-releasing hormone (LHRH) stimulation. Sterility is a rare outcome of mumps orchitis, but it may lead to subfertility (Masarani et al., 2006; Wu et al., 2021).

In the case of HIV, the testis morphology and spermatogenesis are affected with disease progression (Shehu-Xhilaga et al., 2005). The macrophages and CD4^+^ T cells are the main target cells for HIV; these are found in the interstitial space of the testis, but the precise role of the testis in viral shedding during acute HIV infection is not known (Pudney and Anderson, 1991). Several autopsy reports show a high rate of testicular abnormalities in deceased AIDS patients (Pudney and Anderson, 1991). The typical clinical complication of AIDS patients includes oligospermia or azoospermia, orchitis, hypogonadism, and, in some cases, testicular germ cell tumor or lymphoma (Dejucq and Jégou, 2001). Testicular damage and endocrine abnormalities are commonly observed in HIV-positive men (Yoshikawa et al., 1989). Low testosterone levels have been found in men with AIDS. The mode of action of HIV is discussed (Dondero et al., 1996). The virus may facilitate opportunistic infections due to the chronic debilitating illness as part of its indirect effect, but the direct local action was described as responsible for the observed damage within the gonads. The infiltration with lymphocyte and interstitial tissue fibrosis and a decrease in Leydig cell number could result in testosteronemia (Dalton and Harcourt-Webster, 1991; Dejucq and Jégou, 2001).

Before the SARS-CoV-2 pandemic, minimal information about the potential effects of coronaviruses on testis was available. SARS-CoV uses the ACE2 receptor to gain entry into host cells; it is expressed in testicular tissue (discussed in detail in “SARS-COV-2 Pathogenicity and Male Reproductive Tract”) (Harmer et al., 2002). Different reports investigating the testicular specimens of deceased SARS-CoV patients observed the absence of viral particles (Ding et al., 2004; Gu et al., 2005). However, specimens of all seven male patients who tested positive for SARS showed focal testicular atrophy (Gu et al., 2005). In 2006, six cases of deceased SARS patients with orchitis were observed. Testes showing leukocytic infiltration, germ cell destruction, and diminished spermatozoon presence were observed (Xu et al., 2006). In summary, SARS-CoV may affect the testis, and orchitis is the main clinical complication in the male reproductive system.

### Other Tissues Affected by Viremia

Viruses may also be present in semen due to viral replication in the male accessory glands (Salam and Horby, 2017). The testis and epididymis only contribute less than 10% to semen. The seminal vesicles make up 65–75% of the ejaculate volume, and the prostate contributes 25–30%. Although seminal vesicles and the prostate contribute most of the semen volume (Revenig et al., 2014), they have not been not intensively evaluated for RNA infections.

The susceptibility of the seminal vesicles in human males to HIV viral infection both *in vivo* and *in vitro* conditions was examined, showing that seminal vesicles contribute to the viral presence in seminal fluid. Infected cells mainly were seminal vesicle macrophages (Deleage et al., 2011). Moreover, infected cells’ persistence in the seminal vesicles of individuals who received treatment and with no more prolonged detectable viremia signify that the seminal vesicles could be a potential reservoir for many RNA viruses such as HIV (Deleage et al., 2011). The prostate and seminal vesicles of macaques infected with simian immunodeficiency virus (SIV) were profoundly infected reproductive organs, followed by the epididymis and testes *in vivo* (Tortorec et al., 2008).

The prostate secretes necessary fluids for the survival and motility of the sperms. The sexual transmission of ZIKV from a vasectomized male to his partner (Arsuaga et al., 2016) and the long-term presence of ZIKV in the semen of other vasectomized men (Froeschl et al., 2017) have strongly suggested that the prostate and seminal vesicles may operate as potential viral reservoirs for sexual transmission. Another study reported similar results indicating infection, replication, and production of the infectious ZIKV in human prostate cells (Spencer et al., 2017). The hematospermia, dysuria, acute prostatitis, and risk of sexual transmission are potential effects of ZIKV infection on the prostate (Stassen et al., 2018). The seminal fluid is enriched with prostatic secretions and thus be liable for prolonged viral shedding (Revenig et al., 2014) and is also a significant site to anchor chronic infections with diverse pathogens (Dejucq and Jégou, 2001). In conclusion, although seminal vesicles and prostate are generally overlooked when viral infection of the male reproductive tract is studied, it has been demonstrated that RNA viruses can infect those organs and may contribute to viral shedding in semen.

### Sexual Transmissibility of RNA Viruses

Blood–testis barrier and blood–epididymis barrier effectively guard the male reproductive tract, but a broad range of viruses can still breach these barriers and have been identified in semen (Salam and Horby, 2017; Teixeira et al., 2021). The persistence of the virus in semen has several implications. Semen is the leading transporting tool of viruses in sexual transmission; this further implies the potential risk of vertical transmission, embryonic infections, and congenital diseases (Feldmann, 2018). The presence of viruses may also affect semen characteristics and, thus, fertility. It should be noted that merely detecting a virus in semen does not imply that it is sexually transmissible, but epidemiologically, it is a critical public concern and needs to be informed whether the virus can be transmitted sexually, and mainly if the virus can be transmitted sexually by an asymptomatic individual, or fully recovered, or during the infection (incubation period) (Pike et al., 2021; Teixeira et al., 2021).

The prevalence and viral shedding pattern in the semen depend on the type of virus and the viral attachment to specific receptors [summarized in Table 1 and extensively reviewed in Le Tortorec et al. (2020) and Teixeira et al. (2021)]. It is also influenced by several factors such as host characteristics, the levels of viremia, the presence of other pathogens. The viral shedding in semen significantly impacts on the epidemic dynamics. Next, we summarized the current knowledge concerning the presence of virus in semen for the most well-known sexually transmitted viruses: HIV, EBOV, and ZIKV.

The sexually transmitted viruses in humans and their health implications are serious concerns that have grown with the appearance and progression of the AIDS pandemic (Dejucq and Jégou, 2001). Three forms of HIV that are identified in the semen are spermatozoa-associated virions, cell-free virions, and infected leukocytes (Anderson et al., 2010). The free particles or infected cells as virus appear to play an essential role in the transmission; however, spermatozoa are generally accepted to be not productively infected (Dulioust et al., 2002). The development of antiretroviral therapies in combination has been found useful and improving life expectancy in HIV patients. However, the evidence suggests that the immune-privileged nature of testis limits the access of antiviral drugs and antibodies, and viral suppression at the systemic level cannot always be representative of viral suppression in the seminal compartment (Cavarelli and Le Grand, 2020). Interactions between the HIV envelope glycoprotein gp120 and the cell surface receptor CD4 are responsible for the entry of HIV into host cells in most cases, including leukocytes present in semen (Bour et al., 1995; Table 1). CD4 is not found in spermatozoa, but several alternative co-receptors have been explained; Ga1AAg, a glycolipid-related glyceramide, is expressed in ejaculated spermatozoa (30%) and binds with gp120 envelope protein and mannose receptors, identified in only 10% of ejaculated spermatozoa with gp20 binding capacity (Tortorec and Dejucq-Rainsford, 2010; Cardona-Maya et al., 2011). Along with sexual transmission, HIV presence and antiretroviral therapy affect semen parameters (Savasi et al., 2019).

More recently, some reports gained the attention of researchers and health professionals as they indicated that viruses that were previously known for non-sexual transmission (e.g., ZIKV and EBOV) were identified for transmitting the virus sexually to their female partners. These cases of sexual transmission were identified in symptomatically infected patients and disease survivors (Pike et al., 2021). In symptomatically infected men, ZIKV is frequently identified in the semen, and the persistence is much longer than other body fluids, indicating the male genital tract is a reservoir for ZIKV (Mead et al., 2018). The RNA in the semen is not indicative of infectious viral particles, and just fewer samples demonstrated that ZIKV RNA contained in the semen was infectious, suggesting a loss of infectivity of ZIKV RNA in seminal fluid in most of the cases (Musso et al., 2015). The first case of sexual transmission of ZIKV was reported in 2011. Later, the 2014 Zika outbreak in South America reported several ZIKV sexually transmitted cases, further confirming its transmissibility. Recent studies described that the receptor tyrosine kinase AXL is critical for ZIKV entry (Strange et al., 2019; Table 1). Sertoli cells highly express AXL, which is a critical component of the BTB. The immune privilege of the testes promoted their role as ZIKV reservoirs and enabled the sexual transmission of the virus over extended periods. Recent reports claimed that individuals infected with ZIKV have persistence of the virus in the male reproductive tract and semen for a considerable duration even after clinical recovery. The ZIKV infection could cause vertical transmission considering its detection in motile spermatozoa, but more research is needed to verify this route (Silasi et al., 2015). WHO recommends safer sexual practices (using condoms) or abstinence for 6 months for men and 2 months for women returning from active ZIKV transmission areas. This is an effective precaution for most cases, but more research studies are needed to thoroughly understand the sexual transmissibility of ZIKV.

Ebola was first isolated in the 1970s and remained the source of outbreaks in Africa. Two significant Ebola virus disease (EVD) outbreaks took place, including West African and D.R. Congo in 2014 and 2018, respectively. To date, the body of evidence concerning the pathogenesis and epidemiology of EBOV has grown and enhanced. EBOV gains entry into host cells using glycoprotein spikes contained in the viral envelope that achieves the fusion with specific cell receptors at the target cells and subsequent endosome formation (Saeed et al., 2010; Aleksandrowicz et al., 2011). A wide range of cell surface molecules, including integrins, have been described for filoviruses (Table 1). EBOV infection is initiated by virions entering mainly dendritic cells and macrophages. Virus replication in these cells is thought to be critical for initiating systemic infection, spreading the virus to new sites with infection of additional cell populations (Pike et al., 2021).

It has been found that EBOV persists in immunologically privileged sites following clinical convalescence that has occurred because the virus is protected from the immune system of the survivor (Barnes et al., 2017), including areas such as the central nervous system, placenta, and the testes. The viral tropism mechanism for testes remains unidentified. However, it has been speculated that the EBOV manages to persist in the interstitium of the male reproductive tract, including those of prostate gland, seminal vesicle, testis, and epididymis, and is finally transported by infected tissue macrophages to the seminal fluid (Perry et al., 2018). The detection of viral RNA in patients’ semen diminishes gradually after the convalescence; however, the persistence has been accounted to last for 16–18 months (Soka et al., 2016; Deen et al., 2017; Schindell et al., 2018). The majority of the studies that determined EBOV RNA persistence in semen were unable to confirm its infectivity. EBOV sexual transmission has been recorded in the literature. In 2015, a Liberian woman became infected and turned positive for EBOV infection following unprotected sexual intercourse with a male EBOV survivor (Christie et al., 2015). The comparison of viral genome sequence analysis between the survivor and the patient yielded a consistent pattern indicative of direct transmission (Mate et al., 2015). The EBOV particles in the semen survived patients lasted for 179 days from the day of disease diagnosis. Two additional cases of EBOV sexual transmission have been documented, one in Sierra Leone (Arias et al., 2016) and one in Guinea (Diallo et al., 2016). Sexual transmission of Ebola from convalescent survivors is a rare event. However, scientists are still carrying out long-term studies to better understand the effects of EBOV infection particularly the viral persistence and determine effective care and treatment EVD survivors, and consider the importance of sexual transmission in epidemiological models (Brainard et al., 2016; Deen et al., 2017; Fischer et al., 2017).

It is of great importance to study viral shedding in semen, and sexual transmission represents a vital mode of transmission because it has been demonstrated by statistics and mathematical models that this primarily impacts the epidemic dynamics, the size, and the length of the outbreak. It is mandatory to include this information in public health communication campaigns for prevention and control. In the case of HIV, its sexual transmissibility is well known for the general audience. For the recent outbreaks of ZIKV and EBOV, semen is a minor transmission route compared with other modes (i.e., mosquitoes, fomites, other fluids), but scientific evidence confirms it contributes to the epidemic. Even more, the viral shedding in semen for long after patient clinical recovery due to the immune characteristics of the testis may underestimate the potential sexual transmissibility.

## SARS-CoV-2 Pathogenicity and Male Reproductive Tract

### Pathogenic Mechanism of SARS-CoV-2

In December 2019, a new infectious respiratory disease of unknown etiology appeared in Wuhan, Hubei province, China (Zhu et al., 2020). Chinese authorities confirmed on January 7, 2020 that a novel coronavirus was the causative agent, and it was officially named Severe Acute Respiratory Syndrome Coronavirus 2 (SARS-CoV-2) by the International Research Committee on Taxonomy of Pathogens and Viruses (Zhou P. et al., 2020; Zhu et al., 2020). WHO on March 11, 2020 declared Coronavirus Disease 2019 (COVID-19) a pandemic (World Health Organization [WHO], 2020).

SARS-CoV-2 is the seventh described coronavirus known to infect humans (Andersen et al., 2020). In the past two decades, SARS in 2002 and Middle East Respiratory Syndrome (MERS) in 2012 belonging to coronaviruses family were independently responsible for severe pandemics (de Wit et al., 2016). The other described coronaviruses, HKU1, NL63, OC43, and 229E, are associated with mild symptoms (Andersen et al., 2020). Despite the high case fatality rate of SARS-CoV and MERS-CoV, SARS-CoV-2 has caused more infections, deaths, and economic disruptions than these previous coronaviruses.

Coronavirus (CoV) is a single-stranded positive-sense RNA virus with special glycoprotein spikes around the viral envelope, showing a crown-like appearance under an electron microscope (Pal et al., 2020). Coronaviruses have the largest known genomes of animal RNA viruses, ranging from 26 to 32 kb. The nucleocapsid protein (N) forms the capsid outside the genome. The genome is further packed by an envelope that is associated with three structural proteins: membrane protein (M), spike protein (S), and envelope protein (E) (Brian and Baric, 2005). SARS-CoV-2 genome was sequenced (genome size 29.9 kb) and encoded, and contains four structural proteins (S, E, M, and N) and sixteen non-structural proteins (nsp1-16) (Lu et al., 2020).

**FIGURE 2:**
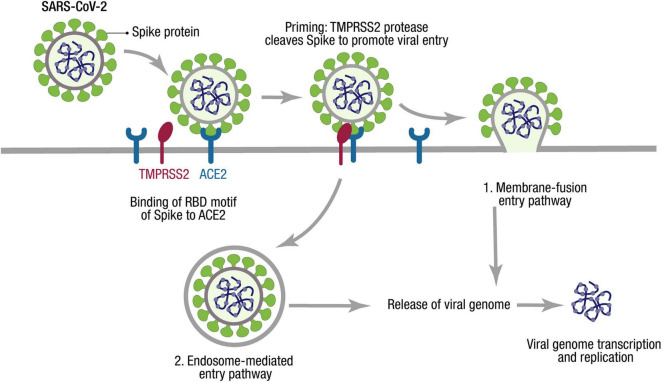
SARS-CoV-2 cell entry mechanism. The SARS-CoV-2 cycle commences by binding the envelope Spike protein to the receptor (ACE2). When recognizing the ACE2 receptor, the RBD stands up to bind the receptor. At the cell membrane, SARS-CoV-2 recruits TMPRSS2, a protease that facilitates viral entry, cleaving S1/S2 site of Spike. Two entry pathways have been described for SARS-CoV-2 cell entry: (1) The presence of proteases triggers the fusion of viral and host membranes. (2) The virus can be endocyted and then released by activation of cathepsin L. Through either entry mechanism, the RNA genome is released into the cytosol, where it is translated and replicated to generate new virions.

To access the host cells, coronaviruses are mediated by spike glycoprotein (S protein), which protrudes from the viral envelope (Figure 2). S protein has two functional subunits (S1 and S2). Viral entry into the target cells depends on the binding of the receptor-binding domain (RBD) in S1 to a cellular receptor, which anchors viral attachment to the target cell surface. In addition, the entry requires S protein priming by cellular proteases, which entails S protein cleavage at the S1/S2 and the S2′ site and allows fusion of viral and cellular membranes (Hoffmann et al., 2020; Huang et al., 2020; Wang M. Y. et al., 2020).

The genomic sequence analysis identified that SARS-CoV-2 is ∼79% identical to SARS-CoV. MERS-CoV is more divergent with only ∼50% nucleotide identity with SARS-CoV-2 (Lu et al., 2020), while SARS-CoV and SARS-CoV-2 identify and use the receptor angiotensin-converting enzyme 2 (ACE2) via the RBD on host cells to enter (Shang et al., 2020a; Figure 2). However, precisely distinct ACE2 interactions enhance higher affinity between ACE2 and SARS-CoV-2 compared with the affinity between ACE2 and SARS-CoV (Shang et al., 2020b).

On the other hand, proteases recruited by SARS-CoV-2 facilitate membrane fusion, especially the transmembrane protease, serine-2 (TMPRSS2), which can cleave S protein, eliminate the structural constraint of S1 on S2, and release the internal membrane fusion peptide, thereby enhancing the viral entry to host cells (Wang M. Y. et al., 2020; Figure 2). The activity of TMPRSS2 is vital to the spread of SARS-CoV-2 and the pathogenesis in the infected host (Hoffmann et al., 2020).

COVID-19 is primarily transmitted through respiratory droplets and contact, and its main symptoms and signs include fever, dry cough, nasal congestion, fatigue, ageusia, lymphopenia, and dyspnea (Wang M. Y. et al., 2020). The disease spectrum of COVID-19 ranges from mild and self-limiting respiratory tract illness to severe progressive pneumonia, multi-organ failure, and death. Recent reports indicate that almost 58% of those infected with SARS-CoV-2 are male, showing that gender was a risk factor for COVID-19 (Zhou F. et al., 2020). Men have been found vulnerable to COVID-19, and thus there are rising concerns about the impact of infectious disease state on male fertility and the possibilities of seminal contamination and transmission.

### Cellular Receptors for SARS-CoV-2 in the Male Reproductive Tract

Tissue and cellular tropism are crucial to understanding the pathogenesis of SARS-CoV-2. The clinical manifestations of COVID-19 strongly correlate with the tissue distribution of the receptor ACE2, a membrane exopeptidase, that is expressed in multiple organ systems (Figure 1). Most viruses infecting humans are inhaled and replicates by utilizing different airway epithelial cells. ACE2 in normal lung tissue is expressed in the nasopharynx and alveolar epithelial cells of the lungs (Harmer et al., 2002; Hamming et al., 2004; Lee et al., 2020). Several gene and protein expression studies have confirmed that ACE2 is also expressed in other tissues such as the intestines, kidney, heart, and testis (Hamming et al., 2004; Jia et al., 2005; Hikmet et al., 2020; Lee et al., 2020; Song H. et al., 2020). COVID-19-related symptoms have been reported in organs expressing high levels of ACE2 (Fan et al., 2020; Olaniyan et al., 2020; Shen et al., 2020).

Different reports have determined the important expression of ACE2 in testis, bringing the reproductive organs into the target radius of SARS-CoV-2 infectivity. It was shown that ACE2 is expressed both in somatic and germ cells, being Sertoli cells the highest ACE2-positive somatic cells followed by Leydig cells, and spermatogonial stem cells, the highest ACE2-positive germ cells (Guo et al., 2018; Hikmet et al., 2020; Liu X. et al., 2020; Wang and Xu, 2020). Also, ACE2 expression was found in the epididymis epithelium and a subset of glandular cells in the seminal vesicle (Hikmet et al., 2020). Physiologically, the expression of ACE2 is negatively correlated with age, and men have higher expression than women of comparable age (He et al., 2020; Zheng et al., 2020).

TMPRSS2 mediates most of the priming of viral spike protein with ACE2. TMPRSS2 is highly expressed with broader distribution, and TMPRSS2 gene transcription is promoted by androgen regulation (Wambier and Goren, 2020). TMPRSS2 has a high expression in the prostatic epithelium and the extracellular vesicles are released into the seminal fluid from the prostate gland at ejaculation, incorporating TMPRSS2 into sperm (Chen et al., 2010; Figure 1). Some authors have indicated that TMPRSS2 is expressed in spermatogonia and Leydig cells (Wang and Xu, 2020), while others indicate that there is almost no overlapping gene expression of ACE2 and TMPRSS2 in human testes (Pan et al., 2020), and that the population on “double-positive” cells constitute only about 0.07% of all prostate epithelial cells (Song H. et al., 2020). Further research investigation is required to appraise the clinical significance of these reports critically.

This evidence suggests that these cells are loci of infection in the male reproductive tract, and sexual transmission might be a part of the transmission routes. Notably, the testes are the immune privilege site, shielding from the inflammatory immune response and harboring an extended presence in the tissue.

### SARS-CoV-2 Effect on the Male Reproductive System

The COVID-19 pandemic has forced the whole scientific community to consider the effect of SARS-CoV-2 on the male reproductive system. As stated before, although the BTB and BEB are tight barriers that reduce the possibility of infection and help to inhibit immune response and testicular inflammation, other RNA viruses can still penetrate the barriers and seen in semen. Also, the specific receptors SARS-CoV-2 uses are expressed in the testis and prostate.

At the time of writing, several research articles have been published regarding the effect of SARS-CoV-2 on the male reproductive tract. It is not the aim of this work to present a comparative analysis of the published results (for systematic review, please see He et al., 2020; Bhattacharya et al., 2021; Pike et al., 2021) but to explore the current information to answer the following: (1) Is SARS-CoV-2 present in semen? (2) Does COVID-19 affect male reproduction directly or indirectly?

Several studies investigated the presence of SARS-CoV-2 in semen, and only two of them have identified the positive detection of virus in semen samples of COVID-19 patients (He et al., 2020). In May 2020, a total of 38 COVID-19 patients’ semen were tested for SARS-CoV-2 by real-time reverse transcriptase-polymerase chain reaction (qRT-PCR), and only six patients were tested positive – of these, two patients were already in the recovery phase, and four patients were still in the active phase of the infection (Li D. et al., 2020). The second report of semen positive for SARS-CoV-2 was published in February 2021. Viral RNA was detected by qRT-PCR in 1 patient out of 15 patients within 0–14 days from the onset of symptom (Machado et al., 2021). In one study report, a total of 15 COVID-19 male patients’ semen were investigated for SARS-CoV-2 (13 patients had mild or moderate disease, and 2 were asymptomatic); the study identified SARS-CoV-2 in one patient semen, but investigators did not specify the patient category or his disease severity (Machado et al., 2021). Furthermore, 14 studies included 262 semen samples from COVID-19 patients that were tested negative for SARS-CoV-2 (Guo et al., 2020; Holtmann et al., 2020; Kayaaslan et al., 2020; Li H. et al., 2020; Ma et al., 2020; Ning et al., 2020; Ozveri et al., 2020; Pan et al., 2020; Paoli et al., 2020; Pavone et al., 2020; Rawlings et al., 2020; Song C. et al., 2020; Ruan et al., 2021; Temiz et al., 2021). Most of the semen samples in these studies were obtained from patients recovering from the disease, and the viremia reported was low and transient.

In summary, two reports indicate the possibility for SARS-CoV-2 shedding into semen, and therefore establish its potential sexual transmission. However, these studies are limited by the small sample size, the lack of specific description for the semen collection modality and detection method (Ct and threshold), and no follow-up of the patients. It is important to note that a positive result was drawn from the detection of viral RNA, which does not effectively reveal the presence of an infective virus (Sethuraman et al., 2020). False-positive results may be obtained as a consequence of the residual urine shedding, as the urinary and genital tract are overlapped in males, or if the sample procurement were not under aseptic conditions, including contamination of RNA fragments from hands or by respiratory droplets (He et al., 2020; Pike et al., 2021). In comparison, the negative results suggest that if the virus ever existed in semen, it was removed by the time of detection in recovery patients. Also, it is possible that if the level of viremia is low and transient, the BTB is successful in protecting from pathogens, and SARS-CoV-2 does not affect the semen (He et al., 2020; Wang W. et al., 2020). In conclusion, to answer our first question, the available evidence to date shows that there is a low but real possibility to find SARS-CoV-2 in semen, explained by the virus tropism and the immune characteristics of the testicular environment. However, it seems unlikely that the virus may replicate in the reproductive tracts, and the location of the virus is only speculative so far. Moreover, there is no evidence of sexual transmission of SARS-CoV-2.

The prostate fluid is one of the essential components of semen, which nourishes and protects the sperm cells. Three studies analyzing 89 prostatic secretion samples of COVID-19 patients were negative for the SARS-CoV-2 RNA by qRT-PCR (Quan et al., 2020; Zhang et al., 2020; Ruan et al., 2021). These reports suggest that prostatic fluid is not affected by SARS-CoV-2, but the sample size is relatively small, and severe COVID-19 patients with high viremia have not been included in any study yet (He et al., 2020; Pike et al., 2021).

It is important to note that the studies investigating semen parameters in COVID-19 patients showed significantly impaired sperm quality (with the decrease in total sperm concentration, reduction in progressive and completely motile sperms, and reduction in a total number of sperm per ejaculate) in moderately infected COVID-19 patients than the patients who recovered from a mild infection and the healthy control group, even though the SARS-CoV-2 RNA could not be detected (Guo et al., 2020; Holtmann et al., 2020; Li H. et al., 2020; Ma et al., 2020; Ruan et al., 2021; Temiz et al., 2021). Fever is one of the common symptoms of COVID-19, and the increased body temperature can impair spermatogenesis. If the body temperature exceeds 38.5°C, even for a limited duration, it could affect the sperm quality for up to 3 months because the ideal temperature of sperm survival is lower than 35°C (2°C lower than the core body temperature) (Ivell, 2007). A significant decrease in the proportion of morphologically normal semen was reported in the COVID-19 patients, and the authors associated this to fever (Ma et al., 2020; Temiz et al., 2021). The results indicated that COVID-19 impaired semen quality in a disease-related manner when the observations were segmented according to the severity of the disease (Holtmann et al., 2020).

Emerging evidence also indicated that SARS-CoV-2 might cause damages to testicular tissues. Similar to SARS-CoV, six studies performed the testicular/epididymal pathological analysis on 33 deceased COVID-19 patients’ reproductive samples, of which they identified 10 of the 33 testicular samples to be positive for SARS-CoV-2 (Barton et al., 2020; Duarte-Neto et al., 2020; Li H. et al., 2020; Yang et al., 2020; Achua et al., 2021; Ma et al., 2021). Furthermore, they also observed germ cells were extensively damaged, Sertoli cell swelling, and significant leukocyte and macrophage infiltration of the testicular interstitium (Li H. et al., 2020; Yang et al., 2020; Achua et al., 2021; Ma et al., 2021). Impaired spermatogenesis was observed in some biopsies (Achua et al., 2021) and testicular atrophy (Barton et al., 2020). Examination of epididymal tissues of six deceased COVID-19 patients also revealed the presence of interstitial edema, congestion, and red blood cell exudation (Li H. et al., 2020). In addition, several studies reported that COVID-19 patients during the acute phase of infection presented scrotal discomfort, with a significantly higher possibility of epididymo-orchitis when the disease was more severe (La Marca et al., 2020; Ozveri et al., 2020; Bridwell et al., 2021; Chen et al., 2021; Ediz et al., 2021).

In the purview of available evidence and to answer our second question about the impact of COVID-19 on male reproduction, it is clear that SARS-CoV-2 can indeed affect testicle function, but this depends mainly on the level of viremia and the specific characteristics of the patients. The clinical presentation is orchitis (Table 1), and it may affect Leydig and Sertoli cell function and sperm quality.

## Perspectives

Some disparate results have been reported so far, but SARS-CoV-2 RNA was undetectable in most semen samples collected from acute patients and patients recovering from COVID-19. This indicates that the risk of contamination of semen with viral particles is low. The sexual transmission of SARS-CoV-2 (as well as SARS-CoV) would be rare and it has not been reported so far, in contrast to other RNA viruses with reported sexual transmission such as HIV, EBOV, HCV, and ZIKV (Table 1). Taking into account in some studies viral RNA has been detected in semen (Li D. et al., 2020; Machado et al., 2021), more research is needed in larger cohorts and to determine whether infective viral particles are present. Antecedent reports suggest that a certain viral threshold may significantly increase the likelihood for the virus to cross the BTB (Liu Y. et al., 2020).

In addition, prior researches suggest that the risk of vertical transmission of SARS-CoV-2 and damage to the embryo via the sperm route is extremely low and has not been reported. However, to reveal the nature of the impact of SARS-CoV-2 on offspring, an effective animal model is required to clarify further if viruses integrate into human sperm and may affect the offspring. One aspect of being considered is that there is growing evidence that perturbations in the environment – such as stress, metabolic alterations, and infection – can induce sperm epigenetic modifications, which can be inherited by the offspring (Champroux et al., 2018; Marcho et al., 2020; Tyebji et al., 2020). It is important to assess if SARS-CoV-2 infection leads to sperm epigenetic alterations. Transcriptional changes due to epigenetic regulation have already been reported for SARS-CoV-2 and SARS-CoV, and MERS-CoV (Salgado-Albarran et al., 2021).

Previous studies have shown that SARS-CoV-2 can have detrimental effects on the male reproductive tract and semen quality and even impair spermatogenesis (Barton et al., 2020; La Marca et al., 2020; Li H. et al., 2020; Ozveri et al., 2020; Yang et al., 2020; Achua et al., 2021; Bridwell et al., 2021; Chen et al., 2021; Ediz et al., 2021; Ma et al., 2021). In most reports, the impact of SARS-CoV-2 on extrapulmonary tissues, including sex organs, was associated with higher viral loads that lead to more severe symptoms and fever (Pike et al., 2021). There is significant variation among studies on the patient characteristics, segmentation, measurements carried out, and so on, making results interpretation difficult (He et al., 2020). From an epidemiological perspective, the significant injury observed in postmortem SARS-CoV-2 patients indicates that SARS-CoV-2 can be present in the male reproductive tract at least for some patients (He et al., 2020; Bhattacharya et al., 2021; Pike et al., 2021). However, only just 10% of the testis showed positive detection for SARS-CoV-2 by RT-PCR (Yang et al., 2020), but it cannot be excluded that these viruses are in blood vessels and not in the testicular cells, in contrast to other pathogeneses in which the viral infection of the reproductive tract is profound and also the viral shedding in sperm (Table 1; Le Tortorec et al., 2020; Teixeira et al., 2021). In this regard, in some cases, the virus may reach the testis because of its cell tropism, as it is the case of HIV, EBOV, and ZIKV. On the other hand, in most cases, the clinical alterations of SARS-CoV-2 on the testicular function may be caused by the systemic “cytokine storm” similar to SARS-CoV cases, H5N1 virus, or influenza.

Conducting a long-term follow-up is essentially critical to determine and screen the reproductive function of patients after recovery. This will help determine and develop standard therapeutic strategies to aid these patients in a timely manner if needed. It would be interesting to have reports involving larger groups with a comprehensive genitourinary examination to discard genitourinary tract infections that may have resulted in testis affections as well.

In SARS-CoV-2–recovered patients, the reproductive potential of most of the patients may not be significantly affected. However, after recovery, some of the patients may later experience infertility issues and reproductive health issues. The existing preliminary results are based on short-term data, as there are no long-term data available on the effects of SARS-CoV-2 on male reproduction. Since the testis is regarded to be an immune privilege site, it is quite possible that the virus may persist for a longer duration in the male reproductive tract as it has been already stated for ZIKV and EBOV (Pike et al., 2021), but less likely for SARS-COV-2, as no evidence had drawn such inference. In the purview of the preliminary investigations conducted, it is essential to continue investigating the adverse health outcomes in patients infected with SARS-CoV-2 upon the reproductive tract.

## Discussion

Over the years, several viruses have been identified causing male reproductive tract infection and persistence and shedding through human semen (Salam and Horby, 2017). The outcomes of these viral infections may be severe and pose a more significant threat not only in terms of possible vertical transmission of the virus or virus-induced mutations to subsequent generations but also for organ integrity, the development of diseases, and changes in the reproductive system and testicular function (Dejucq and Jégou, 2001). HIV is the most studied sexually transmitted virus. Recent outbreaks of ZIKV and EBOV have attracted the scientific community’s interest to understand the possible ways of transmission of the viruses and their clinical implications. The body of research on pathogenesis and epidemiology has expanded over the last years.

The SARS-CoV-2 pandemic has unparallel dimensions in recent history in terms of several infections, deaths, and economic struggles. The scientific community has worked on the characterization of the virus, its clinical implications, diagnosis tools, and vaccines in record time. It is mandatory to understand the nature and mechanism of SARS-CoV-2–host interactions in the male genital tract for its epidemiologic importance and to develop preventive and therapeutic strategies for potential sexual male health implications and sexual transmission of diseases.

## Author Contributions

MIZ and JY performed literature searches on bibliographic databases and wrote the manuscript. MIZ wrote the first draft, revised, edited, and finalized the manuscript. MIZ and HL conceived the topic idea. HL did a critical review of the scientific content of the manuscript. All authors approved the current article for publication.

## Conflict of Interest

The authors declare that they have no competing or conflict of interests to disclose. The funding bodies are public institutions and had no role in study design, conduct, or interpretation of results and its disposition.

## Publisher’s Note

All claims expressed in this article are solely those of the authors and do not necessarily represent those of their affiliated organizations, or those of the publisher, the editors and the reviewers. Any product that may be evaluated in this article, or claim that may be made by its manufacturer, is not guaranteed or endorsed by the publisher.

## Footnotes

1
https://www.worldometers.info/coronavirus/
